# Lipid-chitosan hybrid nanoparticles for controlled delivery of cisplatin

**DOI:** 10.1080/10717544.2019.1642420

**Published:** 2019-07-29

**Authors:** Muhammad Muzamil Khan, Asadullah Madni, Vladimir Torchilin, Nina Filipczak, Jiayi Pan, Nayab Tahir, Hassan Shah

**Affiliations:** a Center of Pharmaceutical Biotechnology and Nanomedicines, Northeastern University, Boston, MA, USA;; b Department of Pharmacy, The Islamia University of Bahawalpur, Bahawalpur, Pakistan;; c Department of Biotechnology, Laboratory of Lipids and Liposomes, University of Wroclaw, Wroclaw, Poland;; d College of Pharmacy, University of Sargodha, Sargodha, Pakistan

**Keywords:** Cisplatin, lipid-polymer hybrid nanoparticles, chitosan, controlled release, ovarian cancer, pharmacokinetics

## Abstract

Lipid-polymer hybrid nanoparticles (LPHNP) are delivery systems for controlled drug delivery at tumor sites. The superior biocompatible properties of lipids and structural advantages of polymers can be obtained using this system for controlled drug delivery. In this study, cisplatin-loaded lipid-chitosan hybrid nanoparticles were formulated by the single step ionic gelation method based on ionic interaction of positively charged chitosan and negatively charged lipid. Formulations with various chitosan to lipid ratios were investigated to obtain the optimal particle size, encapsulation efficiency, and controlled release pattern. Transmission electron microscope and dynamic light scattering analysis demonstrated a size range of 181–245 nm and a zeta potential range of 20–30 mV. The stability of the formulation was demonstrated by thermal studies. Cytotoxicity and cellular interaction of cisplatin-loaded LPHNP were investigated using *in vitro* cell-based assays using the A2780 ovarian carcinoma cell line. The pharmacokinetics study in rabbits supported a controlled delivery of cisplatin with enhanced mean residence time and half-life. These studies suggest that cisplatin loaded LPHNP have promise as a platform for controlled delivery of cisplatin in cancer therapy.

## 1. Introduction

Nanoparticles have shown superiority to other drug delivery systems due to greater solubility for hydrophobic drugs, enhanced circulation time, and targeted drug delivery (Fang et al., 2010). The size gap between endothelial cells in leaky tumor vascular is 100–600 nm, so nanoparticles can reside the tumor site for specific delivery of drugs (Cho et al., 2008). By exploiting the small size of nanoparticles and the unique pathophysiological abnormality of the tumor’s vasculature, the nanoparticles more readily extravasate through the relatively leaky vasculature and release drug at the tumor site. The typical poor lymphatic drainage of the tumor contributes to enhanced retention and accumulation of drug at the tumor site (Acharya & Sahoo, 2011).

A novel lipid-polymer hybrid drug delivery system has been developed to mitigate disadvantages related to liposomal and polymeric nanoparticles systems (Mandal et al., 2013). Hydrophilic biocompatible polymers, integrated with hydrophobic lipid moieties, can self-assemble into nanoparticles. Phospholipids are nonimmunogenic and broadly applied in formulating biocompatible drug delivery systems such as liposomes. However, lipids have stability problem such as degradation at elevated temperature (Robinson, 1996). Combining the polymer and lipid into a single system ensures more effective drug delivery (Elsabahy & Wooley, 2012).

Chitosan is natural cationic polymer with an exceptionally low immunogenicity and greater absorption profile. It also exhibits a facilitated drug release profile in a tumor microenvironment at a low pH (Kumar, 2000). The pH-responsive manner of chitosan makes it suitable as a tumor targeting delivery vector for cancer therapy (Prabaharan, 2015). The lipid component consists mainly of phosphatidylcholine, a natural component of biological membranes, is biocompatible and used in many nanoparticle formulations (Kelmann et al., 2007). The lipid used is LIPOID S75^®^ that contains 74% phosphatidylcholine and is suitable for delivery of drug. Studies have shown an 18% increase in the AUC of drug delivered with such nanoparticles (Fricker et al., 2010). Lipid-chitosan hybrid nanoparticles have the advantage of both polymeric and liposomal drug delivery systems, nanoparticles can be obtained by interaction of positively charged chitosan and negatively charged lipid using an ionic gelation method (see supplementary data) (Barbieri et al., 2013).

Chemotherapy is frequently used for treatment of various forms of cancer. To explore a possible solution to the major drawbacks of chemotherapeutics, including nonspecific and uncontrolled drug delivery, lipid-polymer hybrid nanoparticles were prepared (Tahir et al., 2017). Cisplatin is a first line agent for the treatment of testicular and ovarian cancer and is also used for a number of other malignancies such as lung and esophageal cancer **(**Comis, 1994**)**. However, the poor solubility of cisplatin in water and oil phases limits the development of nanoparticles with high drug loading and encapsulation (Hamelers & De Kroon, 2007). Conventional intravenous administration of cisplatin results in high toxicity in normal tissues particularly liver and kidney. Encapsulation of cisplatin into a lipid-polymer system has resulted in safer delivery to tumors (Kim et al., 2008). Intravenous administration of the cisplatin also leads to rapid clearance due to its low molecular weight (Li et al., 2008a). Application of a lipid-polymer controlled release system can overcome problems associated with low retention time and contribute to improved antitumor efficacy.

This approach for the delivery of cisplatin loaded polymer-lipid hybrid nanoparticles achieved a significantly higher cellular internalization and an enhanced cytotoxic effect compared cisplatin alone. In this study, we employed lipid-chitosan hybrid nanoparticles with high drug entrapment for controlled delivery of cisplatin to tumor cells.

## 2. Material and methods

### 2.1 Materials

Low molecular weight chitosan was obtained from Sigma Aldrich (Chememie, Germany). Lipid (Lipoid S75) was obtained from Lipoid AG (GmbH, Nattermannallee 1, D-50829 Köln, Germany) as a gift sample. Cisplatin was kindly provided by Pharmedic Laboratories PVT (Ltd.) Pakistan. Ethanol and acetic acid were obtained from Fisher Scientific. Cell TiterBlue^®^ was purchased from Promega^®^ (WI, USA). The doxorubicin resistant ovarian cell line A2780 was purchased from Sigma Aldrich. Roswell Park Memorial Institute medium (RPMI), fetal bovine serum (FBS), and penicillin-streptomycin solution were obtained from CellGro (VA, USA). Hoechst 33342 was purchased from Molecular Probes Inc. (Eugene, OR). Paraformaldehyde was from Electron Microscopy Sciences (Hatfield, PA, USA). Trypan blue solution was obtained from Hyclone (Logan, UT, USA).

### 2.2 Preparation of nanoparticles

Nanoparticles were prepared as described by Sonvico et al. (2006). Briefly, 10 mg of chitosan was dissolved in 92 mL of 0.1% acetic acid in deionized water. Cisplatin was dissolved in the same solution with continuous stirring. Lipid was dissolved in pure ethanol (25 mg/mL). The ethanolic solution was then added drop wise to the drug solution. The nanoparticles formed via ionic gelation. The nanoparticles with lipid and chitosan at various ratios were centrifuged at 10,000 rpm for 30 min followed by lyophilization. Six different formulations with lipid: chitosan ratios ranging from 5:1 to 60:1 were characterized for their size, surface charge, entrapment efficiency, and drug loading. In case of cell uptake studies that require incorporation of fluorescent dyes, rhodamine 123 and rhodamine-PE were dissolved in ethanol along with lipid and then ethanolic solution was added drop wise to chitosan solution.

### 2.3 Physicochemical tests

#### 2.3.1 Nanoparticles size and surface charge

The prepared cisplatin-loaded formulations were analyzed for the size, polydispersity index (PdI), and surface charge using a Zeta Sizer (Malvern Ver.7.11 Royston, UK). Dynamic light scattering was used for the determination of size and surface charge. The measurement was performed at 25 °C and a 90° scattering angle in triplicate for each sample.

#### 2.3.2 Determination of drug contents and entrapment efficiency

The entrapment efficiency was calculated by an indirect method by measuring the amount of unentrapped drug in the supernatant after centrifugation (Xu et al., 2006). The drug contents were determined using UV Spectrophotometry (Spectrum Scientific). The absorbance of standard and samples were measured at 210 nm. The determination was performed in triplicate.

#### 2.3.3 Transmission electron microscopy (TEM)

Surface morphology of the cisplatin loaded hybrid nanoparticles was determined by transmission electron microscopy (JEOL USA, Inc.). Sample of LPHNPS with lipid to chitosan ratio 20:1 were applied directly on the grid. The excess of sample was removed from the grid, and suitable images were taken at different magnifications.

#### 2.3.4 Differential scanning calorimeter (DSC)

DSC analysis was performed to evaluate the physical form of cisplatin within the nanoparticles (Alam et al., 2014). The calibration was done using indium for the temperature and heat flow. Samples were placed on one pan and another aluminum pan was used as a reference. The samples were heated over the temperature range of 25–400 °C.

#### 2.3.5 Thermogravimetric analysis (TGA)

Thermogravimetric analysis was performed to measure the change of mass of LPHNPs over a range of temperatures. The dried formulation of chitosan-lipid nanoparticles was used. The LPHNP formulation (38.1 mg) was put on the gravimetric analyzer and the temperature varied from 25 to 500 °C. The change in the mass with the change of temperature was recorded for each second till the completion of run time.

#### 2.3.6 *In vitro* drug release

The drug release study was conducted using a dialysis bag method (Avgoustakis et al., 2002) . The dialysis membrane of MWCO 10–12 kDa was used. The drug release study was performed for all six formulations and the release of the drug from LPHNPs was compared with the pure drug solution. The LPHNPs formulation was suspended in PBS (pH 7.4) in dialysis bags at 37 ± 0.5 °C with constant stirring at 100 rpm. All the formulations were loaded with 5 mg of cisplatin. Samples were withdrawn at predetermined time intervals. Kinetic modeling was applied using zero-order, first-order, Higuchi and Korsmeyer**-**Peppas models.

### 2.4 Biological tests

#### 2.4.1 Cell viability

Cell viability studies were performed on A2780 cells. Cells (5000) were seeded in each well of 96-well plates. After 24 hours incubation, cells were treated with cisplatin-loaded LPHNP and cisplatin solution at a cisplatin concentration range of 1.25 to 50 µg/mL to check the effect at different concentrations (Wang et al., 2014). Formulations were washed out after four hours treatment and replaced with fresh RMPI media. The effect on cytotoxicity was observed at 20 and 44 hours using a Cell TiterBlue^®^ assay measuring fluorescence from cells on plate reader (BioTek).

#### 2.4.2 Fluorescence microscopy

Cells (100,000) were seeded on microscope cover glasses in a 12-well plate. After incubation, cells were treated with the cisplatin loaded LPHNP formulation labeled with Rh-PE and a control blank formulation for 4 h. After four hours, cells were washed with PBS, pH 7.4, and fixed with PBS pH 7.4 containing 2% paraformaldehyde (PFA) for 30 min at room temperature. Cells were then washed with PBS, pH 7.4, three times and stained with 10 µg/mL Hoechst 33342 in PBS pH 7.4 for 15 min. Cells were washed again with PBS, pH 7.4, and mounted on Fisherbrand Superfrost^®^ microscope slides with Fluoromount G^®^ mounting buffer (SouthernBiotech, AL, USA) for analysis by fluorescence microscopy using KEYENCE(BZ-X710) fluorescence microscope.

#### 2.4.3 Cellular uptake

The cellular association of cisplatin loaded LPHNP was evaluated using flow cytometry (Beckton Dickinson FACS Calibur™, NJ, USA). Cells (500,000) were seeded in each well of six-well plates. After overnight incubation, cells were treated with Rh-123 containing formulations (0.5 mol%) for four hours in serum complete media. After that, cells were detached using trypsin and washed with PBS, pH 7.4, three times and centrifuged at 1000 rpm for 5 min and the resuspended in 300 µL of PBS. The fluorescence signal was obtained using a 488 nm laser and the emission was recorded using a 530/30 nm wavelength filter. A total of 10,000 gated live cell events were collected.

#### 2.4.4 *In vivo* pharmacokinetics

Twelve healthy rabbits were selected for the pharmacokinetics studies (average weight, 2.4 ± 0.4 kg) after taking approval from the research and ethics committee on animals of Faculty of Pharmacy and Alternative Medicines, The Islamia University of Bahawalpur (PhD-IUB-Ph-13). Rabbits were obtained from the animal house of Faculty of Pharmacy and Alternative Medicines, The Islamia University of Bahawalpur. Studies support rabbits as an animal model to study pharmacokinetic comparable to human (Nair et al., 1981). Rabbits were divided into two group of six. One group was administered 4 mg/kg of the cisplatin drug solution via an intravenous route as a reference while the other group received 4 mg/kg of the cisplatin loaded lecithin-chitosan hybrid nanoparticles (Huo et al., 2005). Rabbits were fasted overnight before the start of the study. Blood samples were collected at defined time intervals over a period of 24 hours and centrifuged immediately to separate plasma. The plasma samples were treated with nitric acid: perchloric acid to remove plasma proteins (Navolotskii et al., 2015). Plasma was stored at –20 °C. The samples were treated to separate protein and 20 µL samples were used to determine drug concentration using HPLC. HPLC was performed using C18 column with flow rate 1.5 mL/min and UV detector at 250 nm, while acetonitrile/methanol/water was used as a mobile phase in concentration of 30/40/30.

## 3. Results and discussion

### 3.1 Physicochemical tests

#### 3.1.1 Particle size and surface charge

Size and polydispersity index have effects on the loading and release of drug from the nanoparticle formulation (Souza et al., 2014). The particle size and zeta potential of all formulations are shown in (Table 1). The particle size of all developed formulations varied between 181 nm and 245 nm, which is suitable for passive targeting of tumor via an EPR effect (Cho et al., 2008). The surface charge at the 5:1 ratio of lipid-chitosan ratio to a 30:1 ratio remained positive due to the electrostatic interaction of chitosan and lipid. The cationic charge of chitosan prevails. However, as the concentration of lipid was increased, the zeta potential value shifted from positive to negative. At a ratio of 20:1 a monodispersity and a better stability profile were observed because of an interaction of negative and positive charges in appropriate concentration (Sonvico et al., 2006).

**Table 1.** Effect of the lipid to chitosan ratio on particle size(z-average), surface charge, entrapment efficiency, and drug loading.

Lipid: chitosan ratio	Size (nm)	PDI	Surface charge (mV)	EE (%)	DL (%)
5:1	213.0 ± 1.38	0.34	37.1 ± 1.3	83.2 ± 1.2	1.69 ± 0.5
10:1	218.0 ± 0.79	0.43	30.5 ± 2.4	83.8 ± 0.8	1.86 ± 0.6
20:1	181.0 ± 0.43	0.21	21.1 ± 0.8	89.2 ± 0.5	2.07 ± 0.2
30:1	200.9 ± 2.14	0.34	20.5 ± 1.9	89.4 ± 2.3	2.11 ± 0.7

Results indicate average mean ± SD, *n* = 3.

EE: entrapment efficiency; DL: drug loading

#### 3.1.2 Morphology

**Figure 1.**
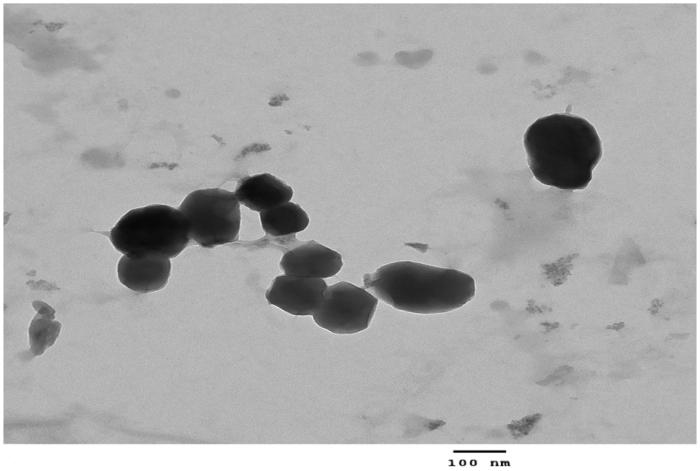
Transmission electron microscope image of lipid-polymer hybrid nanoparticles.

Morphology of the lipid-chitosan nanoparticles was studied using transmission electron microscopy. The TEM images showed the spherical shaped nanoparticles (∼200 nm) with the lipoplex structure of the lipid and polymer entangled with each other through positive and negative charge in the hybrid nanoparticles (Figure 1). The images exhibited a lipoplex morphology with some lipid covering which prevented diffusion of drug and water penetration into the system the shell of chitosan-lipid provides long circulating characteristics (Mandal et al., 2013).

#### 3.1.3 Entrapment efficiency and drug loading

Entrapment of drug in the nanoparticles was calculated by an indirect method. Parameters were evaluated at different concentrations of lipid and chitosan while keeping the concentration of drug constant. All the formulations showed more than 70% encapsulation efficiency. Lipid-polymer nanoparticles at a ratio 20:1 showed the highest encapsulation efficiency (89.2%). The hybrid nanoparticles demonstrated significantly increased encapsulation and drug loading as compared to polymeric nanoparticles, owing to the presence of a lipid layer (Zhang & Zhang, 2010). The presence of a lipid coat on the outer surface of the polymer increased drug encapsulation, and the lipid layer provided structural integrity and prevented leakage of hydrophilic drugs (Cheow & Hadinoto, 2011).

#### 3.1.4 Differential scanning calorimetry

**Figure 2.**
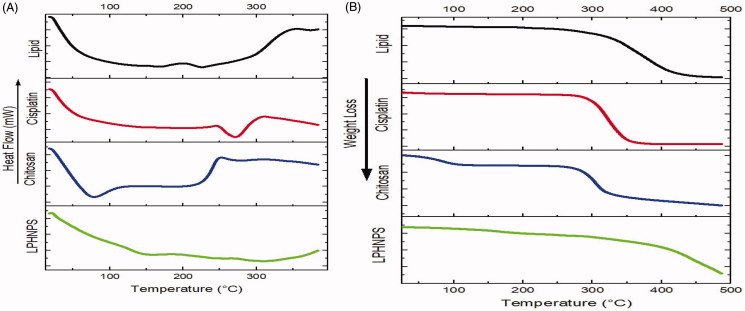
(A) Differential scanning calorimetry graphs of LPHNPs and its individual components against temperature. (B) Thermogravimetric analysis of LPHNPs and its individual components against temperature.

The DSC analysis was performed to check the crystalline/amorphous nature of the drug, polymer and lipid in the formulations (Figure 2(A)). The formulation of cisplatin loaded lipid-chitosan hybrid nanoparticles demonstrated a typical dehydration peak at 102 °C. This is due to the evaporation of water associated with the drug. The cisplatin pure drug showed a dehydration peak at around 100 °C due to the water associated with it and then an endothermic peak at 270 °C, which relates to the melting point of the drug (Dixit et al., 2015). However, there is no sharp endothermic peak of cisplatin in the nanoparticle formulation, attributable to a loss of its crystalline nature in the hybrid nanoparticle formulation, as reported in a previous study (Dixit et al., 2015). The curve of chitosan typically showed a broad endothermic peak over the temperature range of 70–150 °C, corresponding to the loss of water of crystallization and melting point of the chitosan (Cervera et al., 2011).Thermal decomposition of chitosan starts at 300 °C, which is an exothermic process and its peak is seen at around 320 °C (Drebushchak et al., 2006). This behavior of chitosan is also seen in the formulation as an endothermic peak starting at 70 °C and broader endothermic peak in the formulation is due to the presence of chitosan and lipid and cisplatin conversion into an amorphous form.

#### 3.1.5 Thermogravimetric analysis

TGA was performed to measure the percentage of weight loss of LPHNPs over time over a certain range of temperatures (Figure 2(B)). TGA of cisplatin, chitosan, and lipid formulation were also performed. The results show a weight loss by cisplatin occurs at 270 °C, which corresponds to the melting point of cisplatin (Prodana et al., 2014). Chitosan exhibited a slight weight loss at 70 °C and at around 290 °C a gradual weight loss (Javaid et al., 2018). Weight loss in the lipid mixture began at about 250 °C. The melting points of all three components of the formulation were different from each other and showed a peak of weight loss in their respective range confirming the absence of any physical interaction between components and thermal stability of the LPHNPs formulation.

#### 3.1.6 *In vitro* drug dissolution

Drug release studies were performed in phosphate buffer saline pH 7.4 at 37 °C at 100 rpm using the dialysis membrane method (Jeong et al., 2008). None of the formulations demonstrated burst release of cisplatin after immediate immersion in the medium, this is in accordance with previous studies (Spenlehauer et al., 1986). The release pattern of the polymer-lipid hybrid system also showed an absence of immediate burst release and suggested that drug can be released in a controlled manner. There was a sustained release over 24 hours from the lipid-polymer complex (See supplementary data) (Mandal et al., 2016). The results indicated that best fit model for the formulation is a Korsmeyer-Peppas model which is usually followed in lipid-polymer hybrid systems (Tran et al., 2015). The controlled release of drug was attributed to the lipid layer and a minor contribution of polymer and a change in the concentration of lipid effect on the rate of release of the drug from formulations (Chan et al., 2009). The lipid-polymer hybrid nanoparticles with drug distributed inside the polymer showed a better controlled release profile. The release of drug from the polymer matrix depends on diffusion. Further lipid layering prolongs the drug release (Li et al., 2008). The value of release component (*n*) suggests that the drug follows a super case II transport mechanism in most of the formulations (Sonawane et al., 2016). Formulation with 20:1 ratio was selected for further studies.

### 3.2 Biological tests

#### 3.2.1 Cell viability

**Figure 3.**
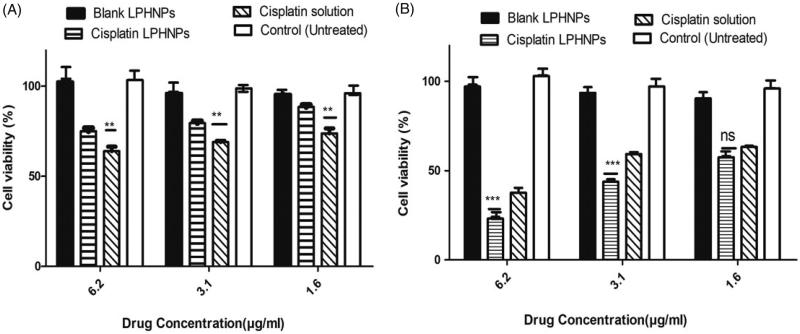
Cytotoxicity profile of cisplatin loaded LPHNPs and drug solution and blank LPHNPs after 24 hr (A) and 48 hr (B). All results indicate mean ± SD, *n* = 3. *p < .05, **p < .01, ***p < .001.

The results were observed using Cell Titer Blue. Six different concentrations of drugs were used 50, 25,12.5, 6.25, 3.12, 1.6 µg/mL. The concentrations with more significant difference are shown in Figure 3. The results showed that blank lipid-polymer hybrid nanoparticles have no effect on cell cytotoxicity and indicate the biocompatibility of the formulation (Figure 3). After 24 hours of incubation the drug solution had greater cytotoxicity to A2780 ovarian cell lines as compared to cisplatin loaded lipid-polymer hybrid nanoparticles. This is due to the drug solution’s easy access to the cell. The drug is in the lipid and polymer layer and takes time to release.

After 48 hours, the cisplatin loaded lipid polymer hybrid formulation had more cytotoxic effect on A2780 ovarian cancer cell lines compared to drug solution, which showed that the release of drug from the formulation could be controlled and produce a greater effect with passage of time. Kim et al. also proved that sustained release of cisplatin from glycol chitosan nanoparticles had higher toxicity compared to cisplatin solution after 48 hours (Kim et al., 2008). Hence the cytotoxic effect of cisplatin is time and concentration dependent. Optimum activity after 48 hours is in agreement with the previous studies (Zhang et al., 2017). It is also important that after 48 hours, the cisplatin loaded lipid-polymer hybrid nanoparticles showed a 20–30% increased cytotoxicity at same concentration as compared to 24 hours and up to 50% of cell death at the lowest concentration. These results are in agreement with the controlled release formulation of cisplatin observed by Reardon et al. (2017). The controlled release of formulation is also evident from the *in vitro* dissolution and *in vivo* pharmacokinetics studies.

#### 3.2.2 Cellular uptake studies

**Figure 4.**
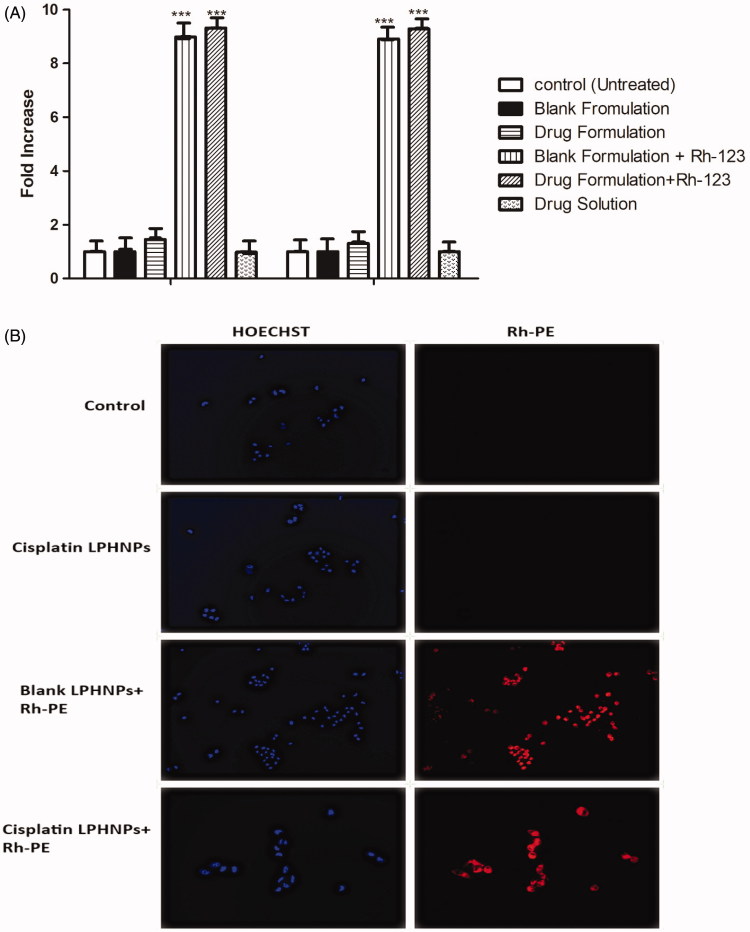
(A) Cell uptake of Rh-123 by the A2780 cell lines. All results indicate mean ± SD, *n* = 3, ***p < .001. (B) Fluorescence microscopy images of the A2780 cell line.

A cell uptake study is an important tool for evaluation of delivery potential of the nanoparticle system. Cell uptake was studied with flow cytometry and cell association by fluorescence microscopy. The results of flow cytometry indicated that there was an eightfold increase in the uptake of cisplatin LPHNPs loaded with fluorescence dye Rh-123 as compared to the control (Figure 4(A)).

The cell association of the nanoparticles was also observed by staining with the fluorescent dyes. Hoechst produces a blue color after interaction with DNA of the cell. Rhodamine PE produces the red fluorescence in cells. The images that nanoparticle show internalization in A2780 ovarian cancer cells. Lipid present in the LPHNP may facilitate the internalization by interacting with the lipid layer at the surface of the cell membrane (Guo et al., 2015). Yue et al. showed that chitosan nanoparticles can deliver drug to the perinuclear space and produce fluorescence (Yue et al., 2011). The cells exhibited an association with LPHNPs and produced fluorescence (Figure 4(B)).

#### 3.2.3 *In vivo* pharmacokinetics

**Figure 5.**
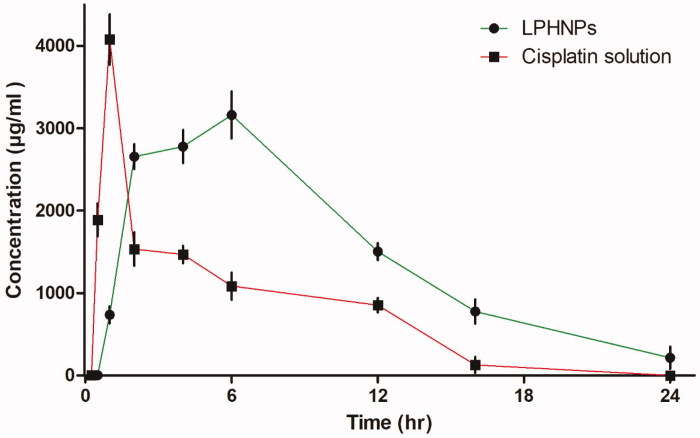
Concentration versus time profile curve of cisplatin LPHNPs and cisplatin solution (mean ± SD, *n* = 6).

To determine the effect of loading cisplatin in lipid-polymer hybrid nanoparticles, analysis of drug was performed by injecting the same amount of drug in two groups of six rabbits. The concentration versus time relationship graph was drawn to evaluate the pharmacokinetics parameters (Figure 5). These studies suggest that combination of polymer and phospholipid can strongly influence the pharmacokinetics properties and could serve as a vehicle for controlled delivery (Feng et al., 2011). The time to reach maximum concentration was 1 ± 0.05 hours in the drug solution group and 6 ± 0.15 hours in the lipid-chitosan hybrid formulation group. Maximum serum concentration was observed at 1.01 mg/mL compared to 4.07 mg/mL in the drug solution group. A lower peak concentration is considered effective for prolonged exposure and reduced side effects of chemotherapeutic drugs (Cheng et al., 2015). The half-life of the cisplatin loaded lipid-chitosan formulation was 14.0 ± 0.4 hours which was much greater than 1.25 ± 0.04 hours for the drug solution group, which indicates a prolonged release of drug in controlled manner. Mean residence time (MRT) was 20.8 ± 0.3 hours compared to 6.0 ± 0.5 hours in cisplatin drug solution, indicating that the lipid-polymer hybrid formulation provides a controlled release of cisplatin. The lipid layer has been observed to protect drug from protein binding and improve absorption (Mei et al., 2013). A similar increase in MRT was observed for a cisplatin formulation by Nakano et al. (1997). The AUC with the lipid-chitosan formulation was 9.83 ± 0.3 mg h/mL as compared to drug solution at 18.4 ± 0.5 mg h/mL. Similarly, the lipid-polymer formulation group shows a 4.6-fold increase in volume of distribution of cisplatin as compared to the cisplatin solution. Kai et al also showed a 4.2-fold increase in volume of distribution of cisplatin (Kai et al., 2015). Overall, the pharmacokinetics parameters of cisplatin were vastly improved with the lipid-polymer hybrid nanoparticle system.

## Conclusion

Cisplatin loaded lipid-chitosan hybrid nanoparticles were successfully fabricated and characterized for various physicochemical parameters including particle size, entrapment efficiency, drug loading and thermal behavior, compatibility of excipients and crystalline behavior and *in vitro* drug release profile. The best formulation with a suitable combination of lipid and chitosan showed monodispersity, small size, and a controlled release profile. Further release of drug was affected by an increase in the lipid concentration. The rate of release of drug was attributable to both polymer and lipid. The release of drug is controlled by the polymer matrix and further by a lipid layer that prevents leakage of drugs. There was an absence of burst release of cisplatin due to its entrapment in the inner polymer layer and outer lipid covering. Cell viability studies confirmed the cytotoxic effect on the A2780 ovarian cancer cell line over a 48-hour period. Cell uptake studies showed increased cellular uptake of LPHNPs. *In vivo* pharmacokinetics studies in rabbits showed a controlled release behavior. Toxicity studies in rats provided safety profile of the LPHNPs. Based on the characterization and invitro release profile, the lipid-chitosan hybrid nanoparticles can provide controlled delivery of cisplatin and act as a useful platform for the potential delivery of cisplatin to tumors. Further studies in tumor animal models should be undertaken to examine the effectiveness of treatment of tumors with LPHNPs.

## Declaration statement

There is no conflict of interest among authors.
